# LncRNA HOTTIP facilitates the stemness of breast cancer via regulation of miR‐148a‐3p/WNT1 pathway

**DOI:** 10.1111/jcmm.15261

**Published:** 2020-04-19

**Authors:** Li Han, Yuanyuan Yan, Lin Zhao, Yinuo Liu, Xuemei Lv, Liwen Zhang, Yanyun Zhao, Haishan Zhao, Miao He, Minjie Wei

**Affiliations:** ^1^ Department of Pharmacology School of Pharmacy China Medical University Shenyang China; ^2^ Liaoning Key Laboratory of Molecular Targeted Anti‐Tumor Drug Development and Evaluation Liaoning Cancer Immune Peptide Drug Engineering Technology Research Center Key Laboratory of Precision Diagnosis and Treatment of Gastrointestinal Tumors Ministry of Education China Medical University Shenyang China

**Keywords:** breast cancer, HOXA transcript at the distal tip (HOTTIP), miR‐148a‐3p, stemness, WNT1

## Abstract

Emerging evidence suggests that dysregulation of long non‐coding RNA (lncRNA) plays a key role in tumorigenesis. The lncRNA, HOXA transcript at the distal tip (HOTTIP), has been reported to be up‐regulated in multiple cancers, including breast cancer, and is involved in various biological processes, including the maintenance of stemness. However, the biological function and underlying modulatory mechanism of HOTTIP in breast cancer stem cells (BCSCs) remains unknown. In this study, we found that HOTTIP was markedly up‐regulated in BCSCs and had a positive correlation with breast cancer progression. Functional studies revealed that overexpression of HOTTIP markedly promoted cell clonogenicity, increased the expression of the stem cell markers, OCT4 and SOX2, and decreased the expression of the differentiation markers, CK14 and CK18, in breast cancer cells. Knockdown of HOTTIP inhibited the CSC‐like properties of BCSCs. Consistently, depletion of HOTTIP suppressed tumour growth in a humanized model of breast cancer. Mechanistic studies demonstrated that HOTTIP directly binds to miR‐148a‐3p and inhibits the mediation of WNT1, which leads to inactivation of the Wnt/β‐catenin signalling pathway. Our study is the first to report that HOTTIP regulates the CSC‐like properties of BCSCs by as a molecular sponge for miR‐148a‐3p to increase WNT1 expression, offering a new target for breast cancer therapy.

## 1 INTRODUCTION

Breast cancer is the most common cancer in women worldwide with high incidence and mortality rates. According to the breast cancer statistics, approximately 271 270 new cases of invasive breast cancer and 42 260 deaths were expected in the United States, in 2019.^1^ Despite the significant advances in therapeutic modalities, including surgery, radiotherapy, and chemotherapy, the mortality rates of breast cancer remain high. Therefore, determining the molecular mechanisms of breast cancer occurrence and progression is crucial for advancing cancer therapies.

Cancer stem cells (CSCs) are a small subpopulation of tumour cells that are involved in self‐renewal, chemoresistance, reoccurrence, and metastasis of cancers.^2^, ^3^, ^4^ Previous studies have shown that breast cancer stem cells (BCSCs) exist ‘hidden’ in breast cancers and can be distinguished by the expression of various biomarkers, such as CD44, CD24, ALDH, EpCAM, and HER2.^5^, ^6^, ^7^ Considering that BCSCs play a key role in tumour initiation and progression, identifying the underlying mechanism of the maintenance of BCSCs will be useful in the development of novel breast cancer‐targeted therapies.

Long non‐coding RNAs (lncRNAs), initially considered to be ‘transcriptional noise’, are a class of functional transcripts longer than 200 nucleotides, with rare protein‐coding capacity.^8^ LncRNAs are not only involved in a variety of biological developmental processes, but are also involved in pathogenesis.^9^ Several studies have shown that dysregulation of lncRNAs may serve as biomarkers for the diagnosis and prognosis of multiple cancers.^10^, ^11^, ^12^, ^13^ Moreover, lncRNAs are potentially associated with cell proliferation, apoptosis, maintenance of stemness, and metastasis of various types of cancers, including breast cancer.^14^ However, few studies have investigated the role of lncRNAs in regulating the stemness of BCSCs. It has been reported that the lncRNA, HOXA transcript at the distal tip (HOTTIP), located at the 5′ end of the HOXA cluster, regulates HOXA gene transcription by directly binding to WDR5 and targeting WDR5/MLL complexes to promote histone H3 lysine 4 trimethylation, and gene transcription.^15^ HOTTIP has also been identified as a cancer‐related lncRNA.^16^ More recently, aberrant HOTTIP expression was reported in hepatocellular carcinoma and pancreatic cancer, among others.^17^, ^18^, ^19^, ^20^, ^21^, ^22^ LncRNA HOTTIP was also reported to be involved in modulating cancer stem cell properties in human pancreatic cancer.^23^ However, whether HOTTIP regulates the stemness of BCSCs and its exact mechanisms remain unclear.

In this study, we aimed to investigate the biological function of HOTTIP in modulating the properties of BCSCs. We found that high expression of HOTTIP was associated with poor prognosis and promoted the stemness of BCSCs. Mechanistic studies revealed miR‐148a‐3p to be a target of HOTTIP. Further, we demonstrated that HOTTIP promoted CSC‐like properties in BCSCs by sponging miR‐148a‐3p. These data may provide a novel target for breast cancer treatment strategies.

## 2 MATERIALS AND METHODS

### 2.1 Cell lines and culture

Human embryonic kidney cells (HEK‐293T), normal human breast epithelial cell line (MCF10A), and breast cancer cell lines (MCF7 and T47D) were obtained from the American Type Culture Collection (ATCC). MCF10A was maintained in Dulbecco's modified Eagle's medium (Gibco) supplemented with 10% foetal bovine serum (FBS). HEK‐293T, MCF7, and T47D cells were cultured in high‐glucose (4.5 mg/mL) DMEM (HyClone) with 10% FBS. MCF7 and T47D sphere cells were maintained in DMEM‐F12 medium supplemented with 2% B27 (Gibco, Thermo Fisher Scientific), b‐FGF (10 μg/L, Promega), and EGF (20 μg/L, Promega). Penicillin‐streptomycin (Life Technologies) was added to all the media to prevent potential contamination.

### 2.2 Lentivirus infection

The lentivirus packaged with plasmids was purchased from Shanghai Genechem Co., Ltd. For lentiviral transfection, cells (2 × 10^5^ cells/well) were seeded in 6‐well plates and grown to 60%‐80% confluence. Next, polybrene (5 μg/mL) and lentiviral vectors were incubated with cells for 24 hours, following which the medium was replaced with fresh medium. After 48 hours of transfection, the cells were treated with puromycin (2 μg/mL) to select for stably infected cells. After 1‐2 generations of selection, the stably infected cells were used for subsequent experiments.

### 2.3 RNA extraction and quantitative realtime polymerase chain reaction (qRT‐PCR)

Total RNA was extracted from the cultured cells using TRIzol (Invitrogen). RNA concentration and purity were analysed using a Nanodrop 2000. cDNA was synthesized using 200‐1000 ng of total RNA, in a 20 µL reaction volume using PrimeScript™ RT reagent Kit with gDNA Eraser (Takara). Realtime PCR analysis was carried out with SYBR^®^ Green Realtime PCR Master Mix (TOYOBO), and the data were normalized to the reference genes β‐actin or U6. Each reaction was performed in triplicate. The relative gene expression level was calculated using the 2^−ΔΔCt^ method.

### 2.4 Protein extraction and Western blot assay

Total protein was extracted using RIPA lysis buffer (Sigma‐Aldrich) supplemented with Halt™ protease inhibitor cocktail (Thermo Fisher Scientific) and Halt™ phosphatase inhibitor cocktail (Thermo Fisher Scientific). The protein concentration was measured using the BCA method. Then the protein samples (20 µg/lane) were denatured at 99°C for 10 minutes and separated by 10% sodium dodecyl sulphate polyacrylamide gel electrophoresis (SDS‐PAGE). They were then transferred to polyvinylidene difluoride (PVDF) transfer membranes (0.45 µm; Millipore, Bedford, MA), followed by incubation with a specific primary antibody. Finally, the membranes were visualized using the Pierce ECL Western blotting Substrate (Thermo Fisher). The primary antibodies used in this study were: OCT4 (1:1000, CST, #2750), SOX2 (1:1000, CST, #3579), β‐actin (1:1000, CST, #3700), CK14 (1:1000, Abcam, ab7800), and WNT1 (1:1000, Abcam, ab15251).

### 2.5 Flow cytometry immunophenotyping assay

The stably infected cells were collected and washed with PBS. Then, the cells were suspended in FACS buffer (00‐4222‐57, eBioscience) and stained with PE‐conjugated anti‐CD24 (311 105, BioLegend) and APC‐conjugated anti‐CD44 (338 805, eBioscience) at 4°C for 30 minutes. After staining, the cells were washed with PBS and resuspended in IC Fixation Buffer (00‐8222‐49, eBioscience) prior to FACS analysis.

### 2.6 Soft‐agar colony formation assay

Culture plates (6‐well) were pre‐covered with a layer of 1.2% SeaPlaque^®^ low melting temperature agarose (Lonza Rockland, ME, USA) in phenol red‐free DMEM medium (SH30284.01, HyClone) supplemented with 20% FBS and 1% penicillin‐streptomycin. A total of 10,000 cells were mixed with 0.6% agarose in phenol red‐free DMEM medium and seeded in the 6‐well plates as the top layer. A total of 1‐2 mL culture medium was added to the surface of solidified agar and the medium was replaced every 3‐5 days. The plates were incubated at 37°C with 5% CO_2_ for 2‐3 weeks. The colonies were then stained with 5 mg/mL MTT, incubated at 37°C for 2 hours, and photographed. Colonies larger than 50 mm in diameter were counted under a microscope.

### 2.7 Mammosphere formation assay

The mammosphere formation assay was performed as described in a previous study with few modifications. Following puromycin selection, the stably infected single cells were suspended in DMEM/F12 (HyClone) supplemented with epidermal growth factor (EGF, 20 ng/mL, Promega), basic fibroblast growth factor (bFGF, 10 ng/mL, Promega), and 2% B27 (Gibco, Thermo Fisher Scientific) and seeded (2000 cells/well) in 6‐well ultra‐low adhesion plates (Corning). The cells were cultured for 7‐14 days and the spheres were imaged. Spheres larger than 100 µm were counted.

### 2.8 Dual‐luciferase reporter assay

The potential miR‐148a‐3p binding sites on HOTTIP were predicted using DIANA (http://diana.imis.athena‐innovation.gr/) tools online system. Fragments containing the wild‐type (WT)/mutated (MUT) sequences of HOTTIP were cloned into the PGL3 luciferase reporter vector, and the resulting reporter constructs were named HOTTIP‐3’UTR‐WT and HOTTIP‐ 3’UTR‐MUT, respectively. Cells were cotransfected with HOTTIP‐3’UTR‐WT or ‐MUT reporter plasmids and Renilla luciferase vector, along with miR‐148a‐3p mimic or miR‐ctrl using Lipofectamine 3000 reagent (Invitrogen). After 48 hours of transfection, luciferase activity was measured using a Dual‐Luciferase Reporter Assay System (Promega) and the data were normalized against Renilla luciferase activity.

### 2.9 Xenograft mouse model

All animal experiments were conducted according to the National Institute of Health Guide for the Care and Use of Laboratory Animals. BALB/c nude mice (4‐6 weeks old) were randomly divided into two groups (n = 5 per group), shControl and shHOTTIP. MCF7 sphere cells stably transfected with shHOTTIP or shControl were suspended in PBS and mixed with Matrigel (BD Biosciences) at a 1:1 ratio. Then, the cells (1 × 10^6^ cells/each mice) were inoculated subcutaneously into the BALB/c nude mice. Following six days of implantation, the tumour volumes were monitored and measured every third day. The tumour volume (V) was calculated as follows: V = length (mm) × width^2^ (mm^2^)/2. After three weeks, the mice were killed and tumours were weighed.

### 2.10 Statistical analysis

Data were analysed with GraphPad Prism 7 and are presented as mean ± standard error of mean (SEM) or mean ± standard deviation (SD), as indicated. For the Western blot results, representative figures from three biological replicates are shown. Densitometric analyses of the bands from the Western blots were performed with ImageJ and normalized to the loading controls. Two‐tailed Student's *t* test was used to compare means between groups as indicated. *P* < .05 was considered statistically significant.

## 3 RESULTS

### 3.1 HOTTIP is highly up‐regulated in breast cancer and BCSCs

**Figure 1:**
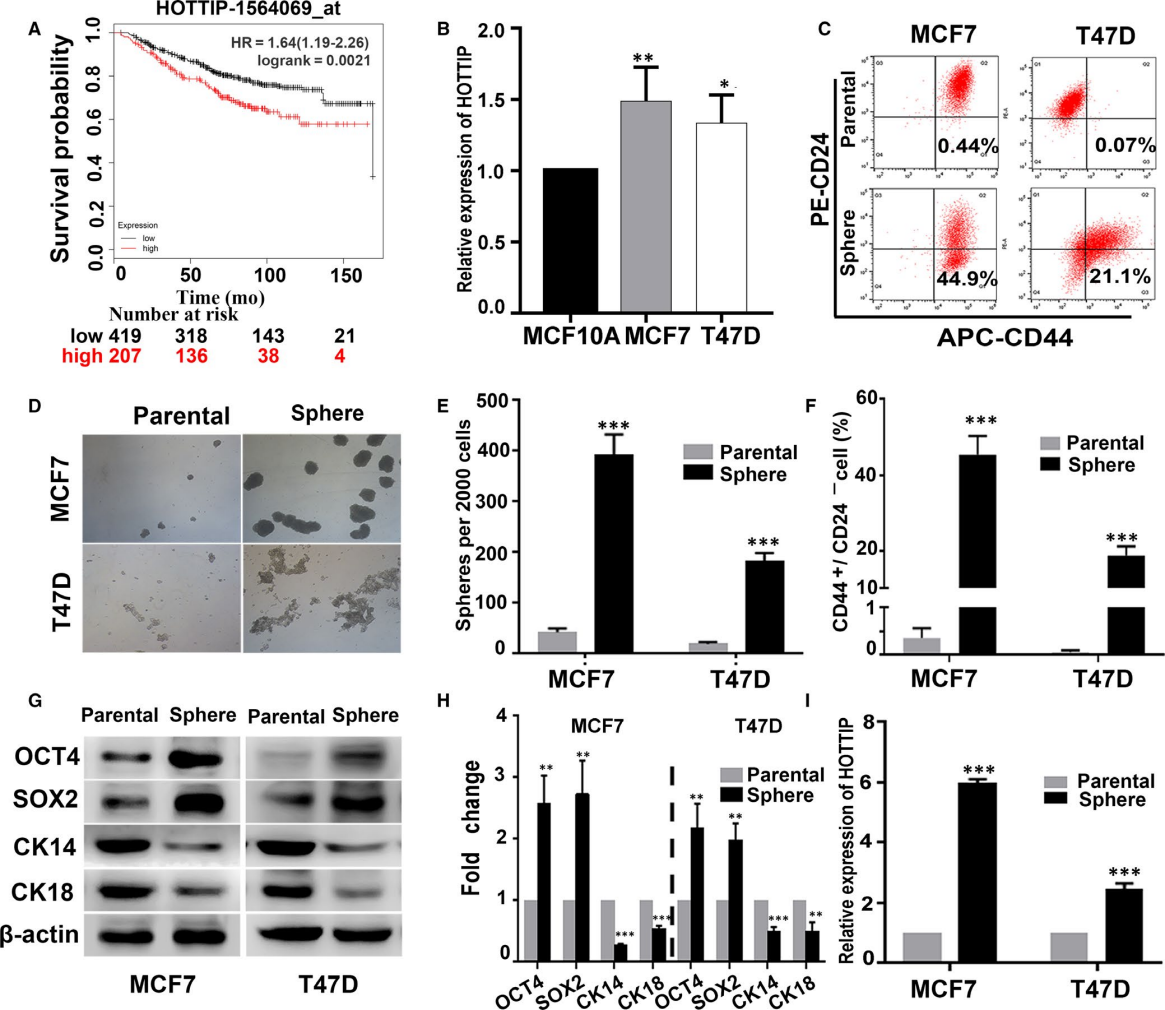
The high expression of HOTTIP in breast cancer and BCSCs. A, The relationship between HOTTIP expression and the outcomes of breast cancer patients was analysed using the online tool, KM plotter (http://www.kmplot.com). B, The expression of HOTTIP by qRT‐PCR analysis in the MCF10A, MCF7 and T47D cells. C, F, The percentage of CD44^+^/CD24^−^ cells by Flow cytometry in the sphere cells of MCF7 and T47D, and their parental cells. D, E, Sphere formation capacities by sphere formation assay in the spheres cells and the parental cells. G, H, Western blot analysis showing the protein expression levels of OCT4, SOX2 and CK14, CK18 in the sphere cells and their parental cells. I, The relative expression of HOTTIP in sphere cells and parental cells was assessed by qRT‐PCR. Data are presented as mean ± SD. *P < .05, **P < .01, ***P < .001 compared to MCF‐10A or the parental cells

It has been reported that HOTTIP expression is significantly increased in breast cancer tissues, compared to adjacent non‐cancerous tissues.^24^, ^25^, ^26^ We used the Kaplan‐Meier plotter (http://www.kmplot.com) to investigate the prognostic significance of HOTTIP by defining upper tertile as cut‐off, and found that patients with high HOTTIP expression displayed shorter overall survival (OS; *P* < .01, Figure 1A). Additionally, we found that the expression of HOTTIP was much higher in MCF7 and T47D breast cancer cells than in MCF‐10A cells (Figure 1B). To determine the expression of HOTTIP in BCSCs, we first enriched for BCSCs (with the CD44^+^/CD24^−^ phenotype) using serum‐free culture media and measured the stemness characteristics of the sphere cells. Flow cytometry showed that the percentage of CD44^+^/CD24^−^ cells was significantly increased in the sphere cells of MCF7 and T47D cells compared to that in the parental cells (Figure 1C,F). Correspondingly, the sphere cells had markedly higher sphere formation capacity in sphere formation assay (Figure 1D,E). In addition, Western blot analysis showed that the stem cell markers, OCT4 and SOX2, were markedly increased, and the luminal epithelial cell markers, CK14 and CK18, were significantly decreased at both the mRNA and protein levels in the sphere cells compared to the parental cells (Figure 1G,H). Furthermore, we found that HOTTIP expression was significantly increased in the sphere cells compared to parental cells (Figure 1I). All these data suggest that HOTTIP may be involved in the regulation of stemness of BCSCs.

### 3.2 HOTTIP is required for maintaining the stemness of BCSCs

**Figure 2:**
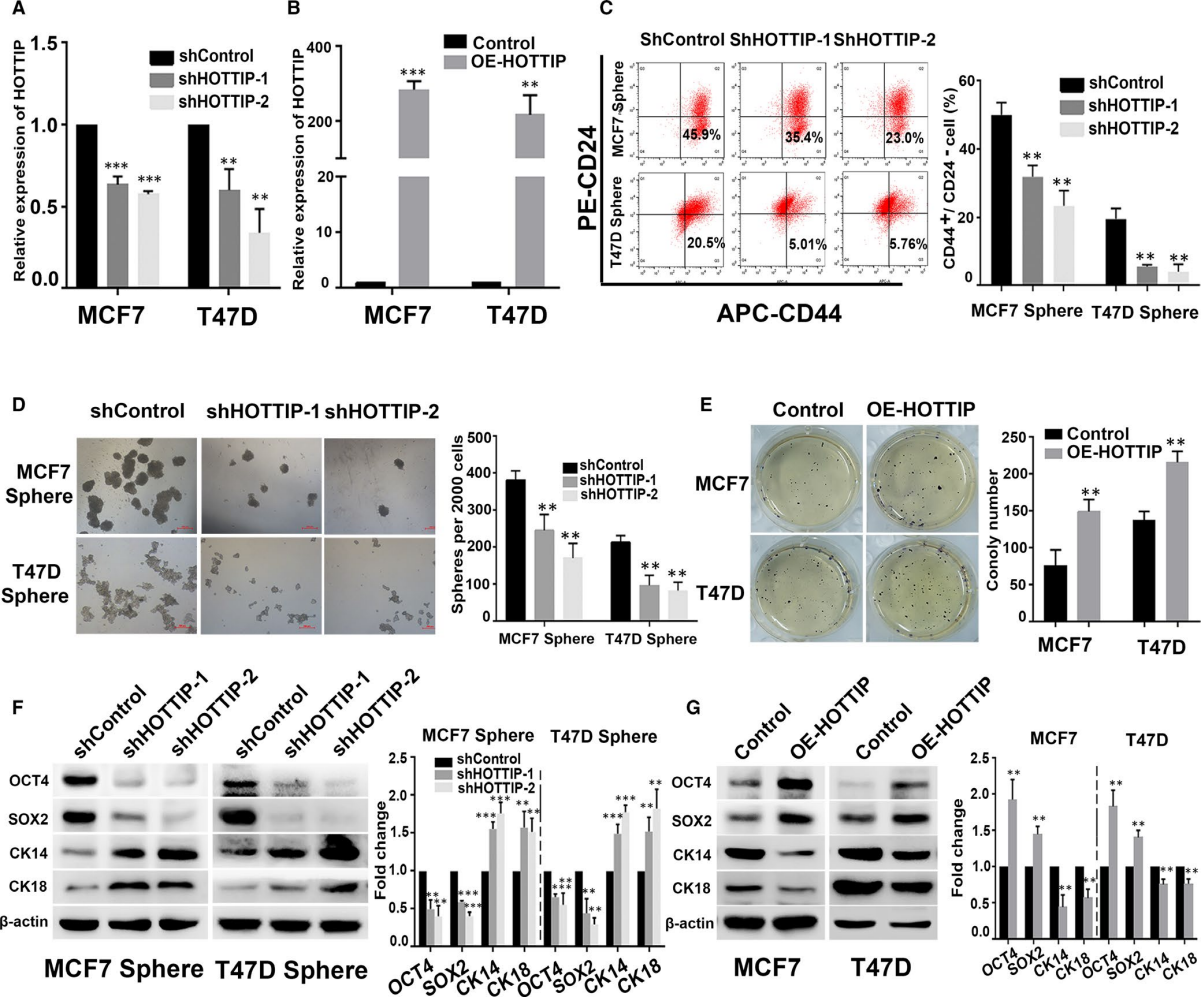
HOTTIP is involved in maintaining the stemness of BCSCs. A,B, The HOTTIP expression was measured in MCF7 and T47D sphere cells transfected with shHOTTIP or shControl plasmids, and in the parental MCF7 and T47D cell lines transfected with overexpression (OE)‐HOTTIP or control plasmids by qRT‐PCR analysis. C, The percentage of CD44^+^/CD24^−^ BCSCs subpopulations was detected by Flow cytometry in the HOTTIP‐silenced MCF7 and T47D sphere cells. D, Sphere formation assays showed the self‐renewal capacity of the HOTTIP‐silenced MCF7 and T47D sphere cells. E, Soft‐agar colony formation assays showed the clone formation ability of the HOTTIP‐overexpressed MCF7 and T47D cells. F, G, The expression of OCT4, SOX2 and CK14, CK18 in the HOTTIP‐silenced MCF7 and T47D sphere cells and in HOTTIP‐overexpressed parental cells by Western blot analysis. Data are presented as mean ± SD. **P < .01, ***P < .001 compared to Control or shControl group

To assess the functional role of HOTTIP in BCSCs, loss‐ and gain‐of‐function studies were performed by in vitro knockdown and overexpression of HOTTIP. The expression of HOTTIP was knocked down in MCF7 and T47D sphere cells by transfecting them with lentiviral plasmids expressing short hairpin RNAs (shRNAs) targeting HOTTIP, shHOTTIP‐1 and shHOTTIP‐2. HOTTIP‐overexpression (OE‐HOTTIP) plasmid was also stably transfected into the parental cell lines, MCF7 and T47D. Following puromycin selection, the transfection efficiency was evaluated by qRT‐PCR. As shown in Figure 2A,B, shHOTTIP markedly decreased the expression of HOTTIP in the sphere cells, whereas the level of HOTTIP was significantly up‐regulated in parental MCF7 and T47D cell lines transfected with OE‐HOTTIP plasmid. We then evaluated the role of HOTTIP in the maintenance of CSC‐like properties using these cells. Flow cytometric analysis revealed that the knockdown of HOTTIP dramatically decreased the population of CD44^+^/CD24^−^ cells in the sphere cells (Figure 2C). In addition, sphere formation assay revealed that the shHOTTIP group had lower self‐renewal capacity compared to the shControl group (Figure 2D). In contrast, the parental MCF7 and T47D cell lines overexpressing HOTTIP showed higher colony formation ability (Figure 2E). Moreover, we performed Western blotting to analyse the expression of OCT4, SOX2, CK14, and CK18. As expected, depletion of HOTTIP effectively down‐regulated the expression of OCT4 and SOX2 and up‐regulated the expression of CK14 and CK18 (Figure 2F). Conversely, overexpression of HOTTIP significantly enhanced the protein levels of OCT4 and SOX2, and markedly decreased the protein levels of CK14 and CK18 (Figure 2G). These data indicate that HOTTIP is involved in the maintenance of stemness of BCSCs.

### 3.3 miR‐148a‐3p is associated with BCSC stemness

**Figure 3:**
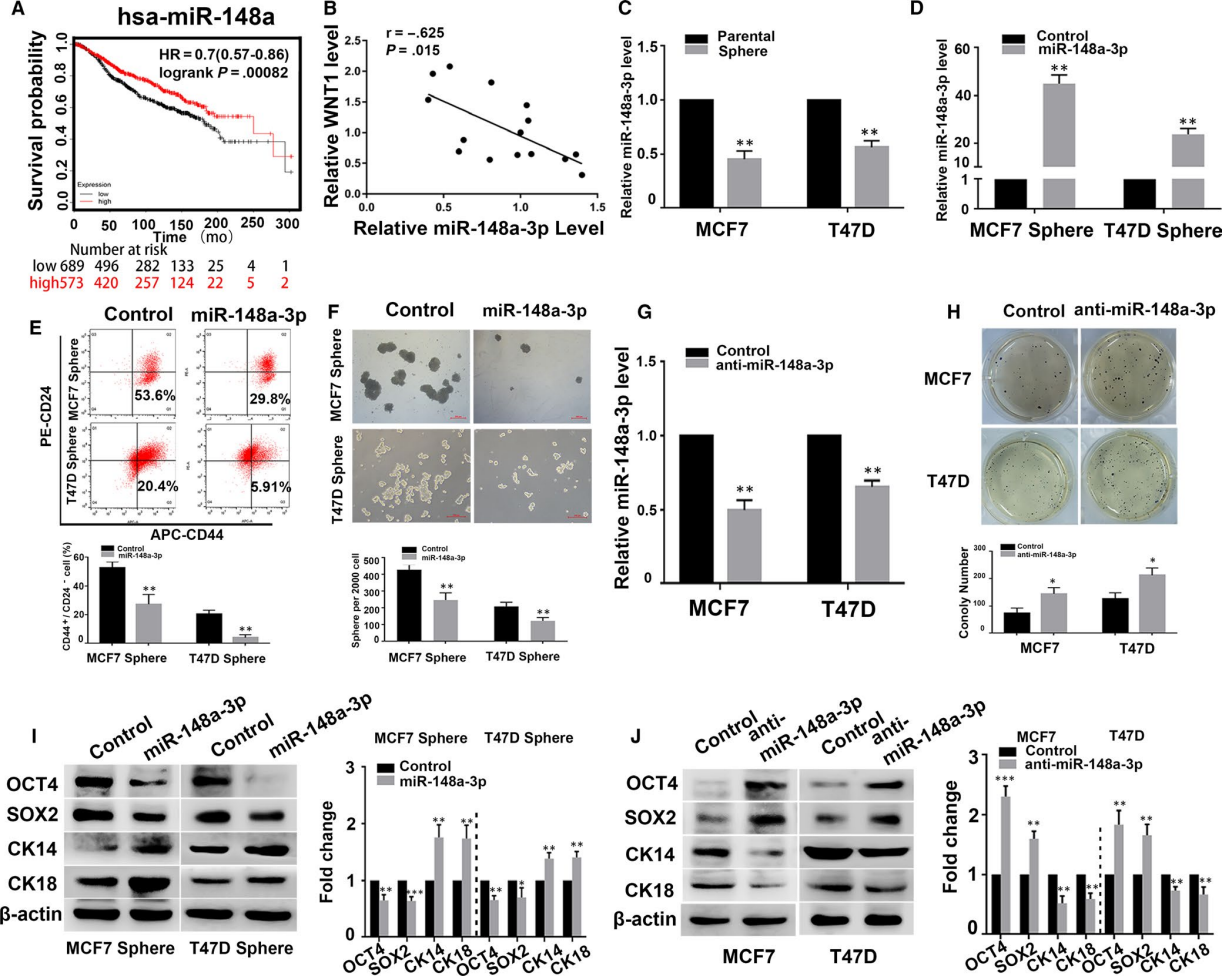
miR‐148a‐3p negatively modulated the stemness of BCSCs. A, The relationship between hsa‐miR‐148a expression and the outcomes of breast cancer patients was analysed using the online tool, KM plotter (http://www.kmplot.com). B, Spearman's correlation coefficient analysis between miR‐148a‐3p expression and WNT1expression in 15 patients with BC. C, The relative expression of miR‐148a‐3p in the sphere cells of MCF7 and T47D, and their parental cells was assessed by qRT‐PCR. D, G, The relative expression of miR‐148a‐3p of the sphere cells transfected with miR‐148a‐3p mimic, and the parental cells transfected with miR‐148a‐3p inhibitor by qPCR analysis. E, The percentage of CD44+/CD24‐ BCSCs subpopulations determined in the miR‐148a‐3p mimic‐transfected sphere cells by flow cytometry. F, The self‐renewal capacity of the miR‐148a‐3p mimic‐transfected sphere cells was analysed by sphere formation assays. H, The clone formation ability of the miR‐148a‐3p inhibitor‐transfected parental cells was detected by soft‐agar colony formation. I, J, The protein levels of OCT4, SOX2 and CK14, CK18 in the miR‐148a‐3p mimic‐transfected sphere cells and the miR‐148a‐3p inhibitor‐transfected parental cells by Western blot. Data are presented as mean ± SD. * *P* < .05, ** *P* < .01 compared to control group

Our previous data showed that miR‐148a expression was decreased in breast cancer.^27^ Kaplan‐Meier analysis also showed that patients with low miR‐148a expression experienced worse survival (*P* < .001, Figure 3A). We also detected the expression of miR‐148a‐3p and WNT1 and found that the expression of miR‐148a‐3p was negatively correlated with that of WNT1 (Figure 3B). In this study, we found that MCF7 and T47D sphere cells had lower miR‐148a‐3p expression than the parental cells (Figure 3C). To investigate the function of miR‐148a‐3p in BCSCs, MCF7 and T47D sphere cells were transfected with Lv‐miR‐148a‐3p mimic while the parental cells were transfected with the Lv‐miR‐148a‐3p inhibitor. As shown in Figure 3D,G, qRT‐PCR assay revealed that the miR‐148a‐3p mimic dramatically increased the expression of miR‐148a‐3p in the sphere cells, whereas miR‐148a‐3p inhibitor significantly down‐regulated the expression of miR‐148a‐3p in the parental cells. We also found that the percentage of CD44^+^/CD24^−^ cell subpopulation was decreased in the miR‐148a‐3p mimic‐transfected sphere cells compared to the control transfected cells (Figure 3E). In MCF7 and T47D cells, sphere formation capacity was significantly decreased in the miR‐148a‐3p mimic group than in the control group (Figure 3F). In contrast, inhibition of miR‐148a‐3p greatly enhanced the colony formation capacity of MCF7 and T47D cells (Figure 3H). In addition, miR‐148a‐3p overexpression resulted in a significant down‐regulation of OCT4 and SOX2 expression, and up‐regulation of CK14 and CK18 expression (Figure 3I). Conversely, miR‐148a‐3p inhibition caused a significant up‐regulation of OCT4 and SOX2 expression and down‐regulation of CK14 and CK18 expression (Figure 3J). Taken together, these functional studies indicate that miR‐148a‐3p may participate in the regulation of the stemness of BCSCs by playing an opposite role to HOTTIP.

### 3.4 HOTTIP directly interacts with miR‐148a‐3p

**Figure 4:**
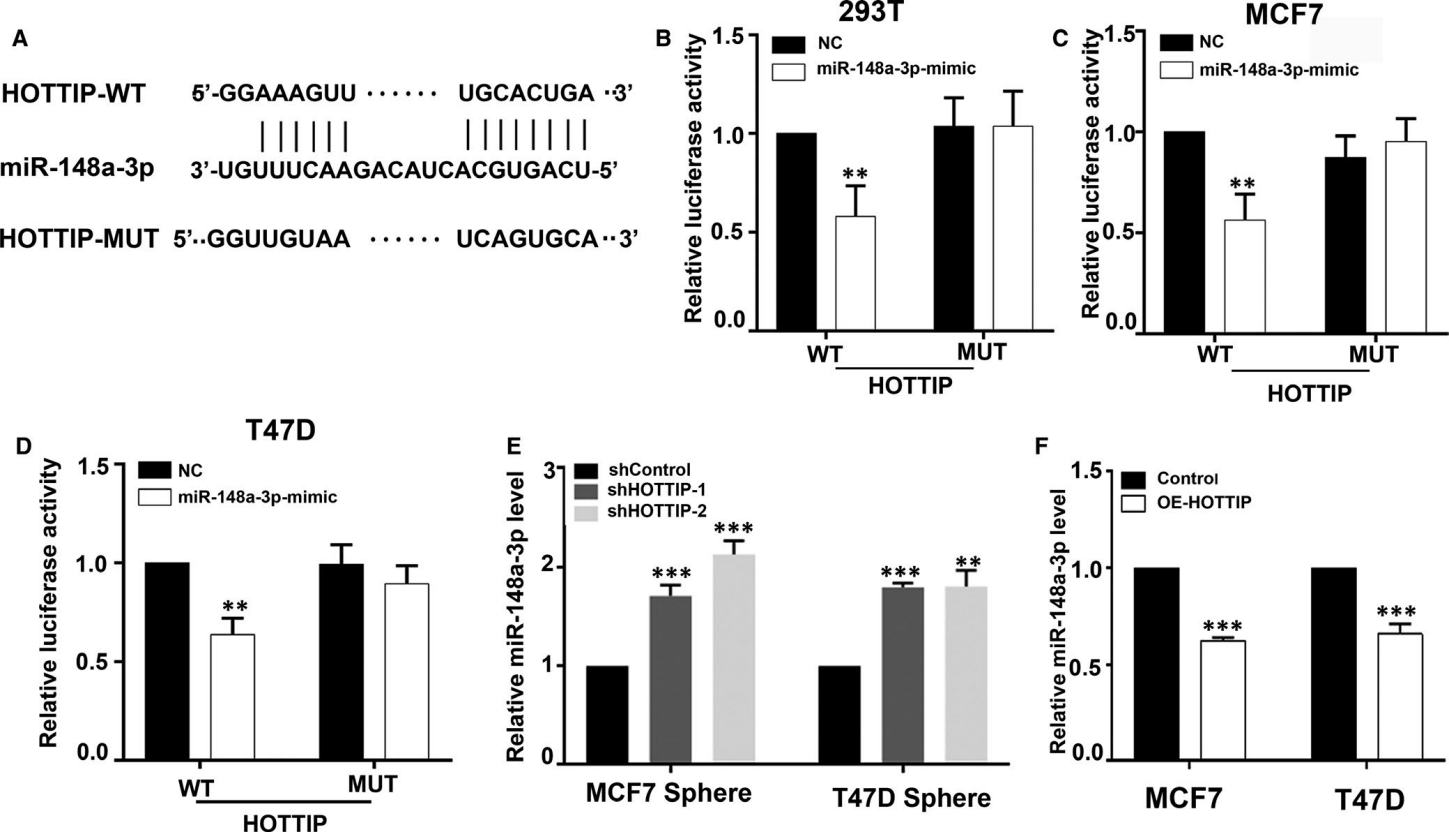
HOTTIP regulates miR‐148a‐3p by acting as a ceRNA. A, A putative binding site between HOTTIP and miR‐148a‐3p by DIANA TOOLS. B, C, D, Luciferase reporter assay in the HEK‐293T, MCF7 and T47D cell lines cotransfected with WT and MUT type HOTTIP reporters and miR‐148a‐3p mimics. E, F, qRT‐PCR was performed to test the expression of miR‐148a‐3p in the sh‐HOTTIP‐transfected sphere cells of MCF7 and T47D, and the OE‐HOTTIP‐transfected parental cells. Data are presented as mean ± SD. **P < .01, ***P < .001 compared to control or shControl group

Considering the opposite roles of miR‐148a‐3p and HOTTIP in the regulation of the stemness of BCSCs, we sought to determine whether HOTTIP functioned as a ceRNA by binding to miR‐148a‐3p. We first use DIANA tools to perform bioinformatics prediction analysis. The putative binding sites between miR‐148a‐3p and HOTTIP are shown in Figure 4A. To validate the direct binding between miR‐148a‐3p and HOTTIP, we generated luciferase plasmids carrying HOTTIP wild‐type (HOTTIP WT) and HOTTIP mutation (HOTTIP MUT). The results showed that miR‐148a‐3p overexpression significantly decreased the luciferase activity of HOTTIP WT, while it had no effect on the luciferase activity of HOTTIP MUT in HEK‐293T cells (Figure 4B). Similar results were observed in MCF7 cells and T47D cells (Figure 4C,D). Moreover, we observed that the depletion of HOTTIP markedly increased miR‐148a‐3p expression in the sphere cells of MCF7 and T47D cells (Figure 4E), while the overexpression of HOTTIP markedly reduced miR‐148a‐3p expression in the parental cells (Figure 4F). Taken together, we speculate that HOTTIP may regulate miR‐148a‐3p by acting as a ceRNA.

### 3.5 HOTTIP modulates the stemness of BCSCs through miR‐148a‐3p/WNT1 signalling

**Figure 5:**
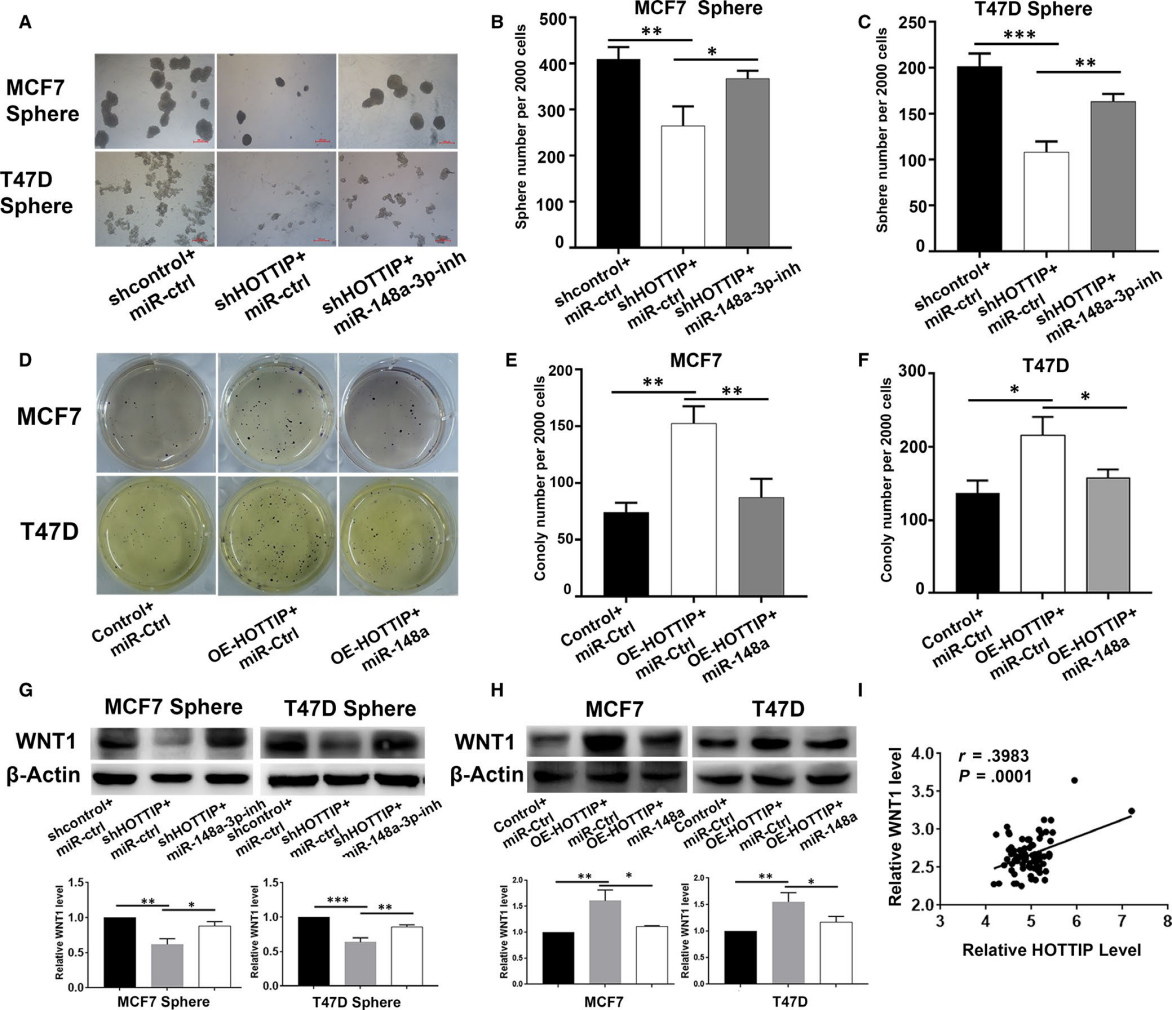
HOTTIP modulates the stemness of BCSCs via miR‐148a‐3p/WNT1 signaling. A, C, The effects of miR‐148a‐3p inhibitor on the self‐renewal capacity of the shHOTTIP‐transfected MCF7 and T47D sphere cells by sphere formation assays. B, D, The effects of miR‐148a‐3p mimic on the self‐renewal capacity of the OE‐HOTTIP‐transfected MCF7 and T47D parental cells by soft‐agar colony formation. E, The effects of miR‐148a‐3p inhibitor on the WNT1 protein levels of the shHOTTIP‐transfected MCF7 and T47D sphere cells by Western blot assay. F, The effects of miR‐148a‐3p mimic on the WNT1 protein levels of the OE‐HOTTIP‐transfected MCF7 and T47D parental cells. G, The correlation between HOTTIP and miR‐148a‐3p was analysed by Spearman's correlation analysis. H, The relationship of HOTTIP and WNT1 expression was analysed by Spearman's correlation analysis. I, GEO dataset revealed a significant positive correlation between HOTTIP and WNT1 expression. Data are presented as mean ± SD. *P < .05, **P < .01, compare with Control or shControl group

To further explore whether HOTTIP modulates the stemness of BCSCs by regulating miR‐148a‐3p, rescue experiments were performed. We set up three groups as shControl, shHOTTIP, and shHOTTIP + miR‐148a‐3p inhibitor. We found that the inhibitory effects of depletion of HOTTIP on the self‐renewal capacity of MCF7 and T47D sphere cells were reversed by the miR‐148a‐3p inhibitor in sphere formation assays (Figure 5A,C). Conversely, the miR‐148a‐3p mimic abolished the increase in the self‐renewal capacity of parental cells following HOTTIP‐overexpression (Figure 5B,D). Our previous study showed that miR‐148a inhibited breast cancer migration and invasion by directly targeting WNT1. Therefore, we further explored the relationship between HOTTIP and WNT1. Western blot analysis showed that the protein level of WNT1 was significantly decreased in shHOTTIP‐transfected sphere cells compared to control cells. However, the results were reversed by the miR‐148a‐3p inhibitor (Figure 5E). In contrast, overexpression of HOTTIP significantly increased the expression of WNT1, which was abolished by miR‐148a‐3p overexpression (Figure 5F). In order to investigate the association between HOTTIP, miR‐148a‐3p and WNT1, we detected their expression in clinical specimens. We found that HOTTIP levels were negatively correlated with miR‐148a‐3p levels in BC tissues (Figure 5G) and the expression of HOTTIP was positively correlated with that of WNT1 (Figure 5H). In addition, we used the GSE6532 dataset to explore the relationship between HOTTIP and WNT1. The results showed that the level of HOTTIP was positively correlated with WNT1 (Figure 5I). These results suggest that HOTTIP modulates the stemness of BCSCs by regulating the WNT1 pathway by acting as a ceRNA and sponging miR‐148a‐3p.

### 3.6 HOTTIP facilitates tumorigenesis of breast cancer in vivo

**Figure 6:**
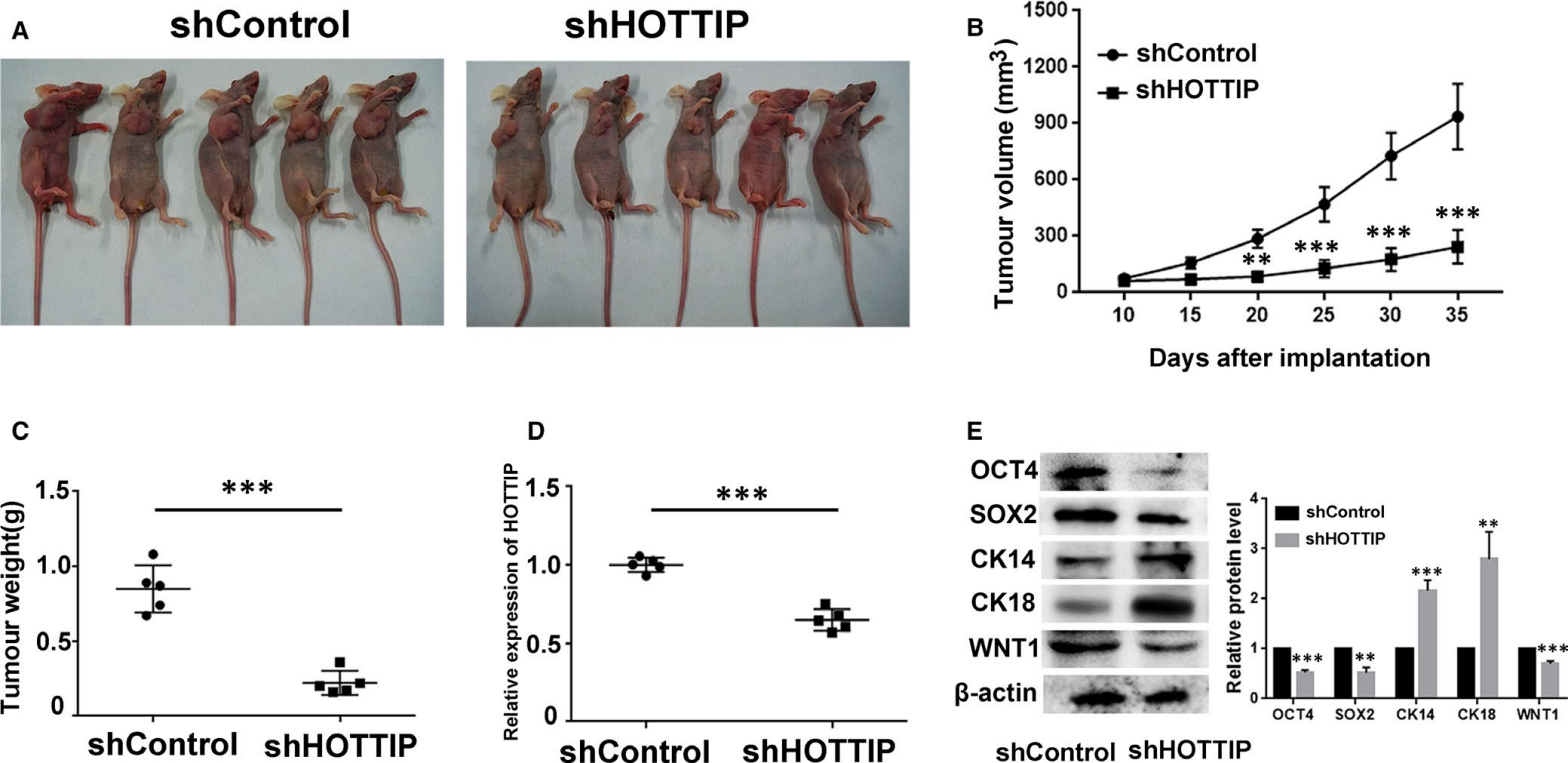
Effect of HOTTIP knockdown on tumour growth. A, Image of tumour formation from HOTTIP‐silenced group and negative control group at the end of point. B, Tumour growth curves were plotted for HOTTIP‐silenced group and negative control group by the tumour volume. The tumour volume was calculated every three days. C, Comparison tumour weight between HOTTIP‐silenced group and negative control group. D, The expression of HOTTIP in HOTTIP‐silenced group and negative control group was determined by qRT‐PCR. E, OCT4, SOX2, CK14, CK18, and WNT1 expressions in HOTTIP‐silenced group and negative control group were detected by Western blot. Data are presented as mean ± SD. **P < .01, ***P < .001, compare with shControl group

To further validate the oncogenic effect of HOTTIP in vivo, we carried out animal experiments. MCF7 sphere cells with shControl and shHOTTIP were subcutaneously injected into nude mice. As shown in Figure 6A–C, the depletion of HOTTIP decreased the volume and weight of the xenograft tumours, compared to the shControl group. In addition, qPCR analysis confirmed the knockdown efficiency of HOTTIP (Figure 6D). We analysed the expression of OCT4, SOX2, CK14, CK18, and WNT1 in the tumour tissues. The levels of OCT4, SOX2, and WNT1 decreased following the silencing of HOTTIP, while the levels of CK14 and CK18 increased (Figure 6E). These results indicate that HOTTIP promotes tumorigenesis of breast cancer in vivo.

## 4 DISCUSSION

Breast cancer is well recognized as the most common malignancy in women worldwide.^1^ Although the treatment options currently available for breast cancer patients, including surgery, chemotherapy, endocrine therapy, and radiation therapy, have improved,^28^ the mortality rate associated with breast cancer remains high. Therefore, better understanding of the underlying mechanisms and molecules involved in tumorigenesis and progression is critical to search for potential therapeutic targets to improve the survival of breast cancer patients. CSCs are defined as a small population of cancer cells, similar to progenitor cells, which possess the capacity for self‐renewal, self‐differentiation, and tumorigenicity.^2^ Several studies have confirmed the identification and isolation of CSCs from multiple solid tumours, such as breast cancer, lung cancer, colorectal cancer, liver cancer, glioma, and ovarian cancer.^5^, ^29^, ^30^, ^31^, ^32^, ^33^ CD44^+^/CD24^−^ BCSCs were first isolated from breast cancer in 2003.^5^ Given the characteristics of BCSCs, specifically targeting BCSCs may be a promising therapeutic strategy for breast cancer.

Increasing number of studies have demonstrated that lncRNAs play a vital role in modulating the stemness of CSCs.^34^ For example, lncTCF7 is highly expressed in HCC tumours and liver CSCs and promotes self‐renewal of liver CSCs by mediating Wnt signaling.^35^ Up‐regulation of Linc00152 promotes malignant progression of glioma stem cells by regulating the miR‐103a‐3p/FEZF1/CDC25A pathway.^36^ LncBRM drives self‐renewal of liver CSCs by activating YAP1 signaling.^37^ HOTTIP, also known as HOXA‐AS6, HOXA13‐AS1, and NCRNA00213, maps to chromosome 7p15.2, and encodes a non‐coding RNA (ncRNA) of ~4 kb. Several studies have demonstrated that HOTTIP is highly expressed in multiple cancers, including liver, kidney, lung, colorectal, and pancreatic cancer, and is involved in tumour progression.^17^, ^18^, ^38^, ^39^, ^40^ For example, HOTTIP plays an oncogenic role in small cell lung cancer by acting as a ‘sponge’ to bind miR‐574‐5p.^39^ Moreover, HOTTIP is reported to be associated with disease progression and predicts the outcome in hepatocellular carcinoma patients.^17^ In addition, HOTTIP can also mediate HOXA9 to enhance the stemness of pancreatic cancer cells.^23^ However, the role of HOTTIP in BCSCs has not been reported. In the current study, we showed that HOTTIP modulates the CSC‐like characteristics of BCSCs by regulating the miR‐148a‐3p/WNT1 axis.

Consistent with previous studies, we found that HOTTIP is highly expressed in breast cancer cells. We used serum‐free medium to enrich for CD44^+^/CD24^−^ BCSCs. qRT‐PCR results showed that the expression of HOTTIP was significantly increased in the sphere cells compared to parental cells. We also verified that HOTTIP was associated with poor outcome in breast cancer patients using Kaplan‐Meier analysis. These results indicated that HOTTIP plays an oncogenic role in breast cancer. To identify the biological role of HOTTIP in BCSCs, gain‐ and loss‐of‐function assays were conducted. The results showed that depletion of HOTTIP decreased the subpopulation of stem cells, limited sphere formation and expression of stem factors (OCT4 and sox2), promoted the expression of differentiation markers (CK14 and CK18), and suppressed tumorigenesis in vivo. In contrast, up‐regulation of HOTTIP significantly promoted colony formation, and the expression of OCT4 and SOX2, and inhibited the expression of CK14 and CK18. Collectively, these data demonstrate that HOTTIP may play a critical role in maintaining the stemness of BCSCs.

Increasing number of studies have shown that lncRNAs can act as endogenous molecular sponges and sequester miRNAs to modulate target gene expression.^41^ To determine the mechanism of HOTTIP function in BCSCs, we used the DIANA tool to find the potential target of HOTTIP.^42^ The results showed that miR‐148a‐3p was a putative target of HOTTIP. A dual‐luciferase reporter gene assay confirmed that HOTTIP directly bound to miR‐148a‐3p. It has been reported that miR‐148a is involved in cancer development and progression.^43^ Expression of miR‐148a is significantly down‐regulated in liver cancer and miR‐148a acts as an inducer of hepatocytic differentiation.^44^ MiR‐148a was reported to sensitize cells to TRAIL and reduce lung tumorigenesis by down‐regulating MMP15 and ROCK1.^45^ We also previously reported that miR‐148a suppressed breast cancer migration and invasion by inhibiting WNT1.^27^ In this study, we demonstrated that miR‐148a‐3P was dramatically decreased in BCSCs and could be regulated by HOTTIP to suppress the stemness of BCSCs.

Previous studies have shown that the Wnt/β‐catenin pathway plays a critical role in the development and progression of cancer.^46^ In addition, the Wnt/β‐catenin pathway is also involved in the maintenance of CSC function.^47^ WNT1 is a key molecule in the Wnt/β‐Catenin pathway. Rescue experiments demonstrated that HOTTIP acted as a ‘sponge’ of miR‐148a‐3p to modulate WNT1, which could trigger the Wnt/β‐Catenin pathway.

## CONFLICTS OF INTEREST

All authors declare that they have no competing interests.

## AUTHOR CONTRIBUTIONS

LH, MH and MJW conceived the study design. LH, YYY, YNL, XML, YYZ, LWZ, and HSZ performed experiments and collected all data. LH, LZ, MH and MJW collected and analysed data and drafted the manuscript. LH, MH and MJW assisted in drafting and revising the manuscript. All authors read and approved the final manuscript.
